# 857. Impact of Rapid Antibiotic Susceptibility Testing for Gram Negative Bacteremia Varies by Pathogen Type and Resistance: A Secondary Analysis of the RAPIDS GN Trial

**DOI:** 10.1093/ofid/ofad500.902

**Published:** 2023-11-27

**Authors:** Ritu Banerjee, Abhigya Giri, Lauren Komarow, Maria Souli, Sarah B Doernberg, Robin Patel

**Affiliations:** Vanderbilt University Medical Center, Nashville, TN; The George Washington University, Rockville, Maryland; George Washington University, Rockville, Maryland; Duke University, Durham, North Carolina; University of California, San Francisco, San Francisco, California; Mayo Clinic, Rochester, MN

## Abstract

**Background:**

The RAPIDS GN trial, a prospective randomized controlled trial, showed that rapid blood culture antibiotic susceptibility testing (AST) led to faster antibiotic therapy changes compared to standard of care (SOC) testing for Gram negative bacteremia (Banerjee et al, Clin Infect Dis (2021) 73:e39-e46). A secondary analysis of the data was conducted to assess impact of rapid testing on appropriateness of antibiotic spectrum by pathogen and resistance profile.

**Figure:**
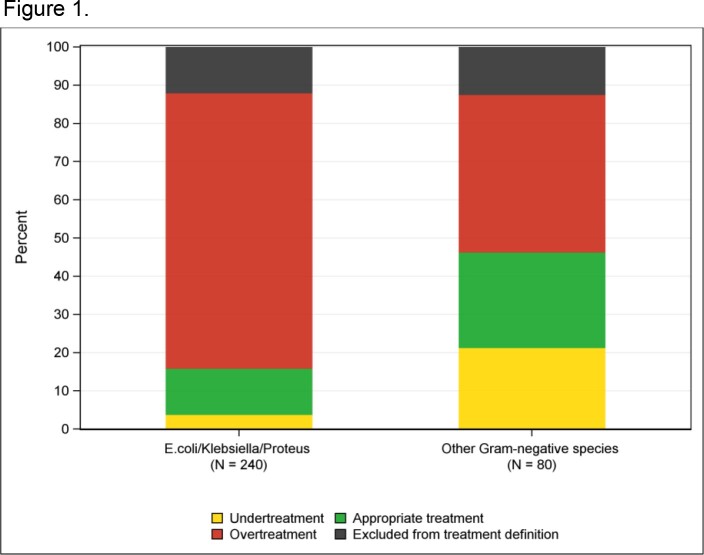

Empiric treatment (0 to 2 h post randomization) among subjects with antibiotic-susceptible isolates of monomicrobial, on-panel Gram negative species (N = 320). For this analysis subjects from both SOC and rapid testing groups were included. Black: Excluded from treatment definition if subjects received no antibiotics, antibiotics without susceptibility results reported, or only an aminoglycoside, azithromycin, penicillin, rifaximin, or atovaquone.

**Figure:**
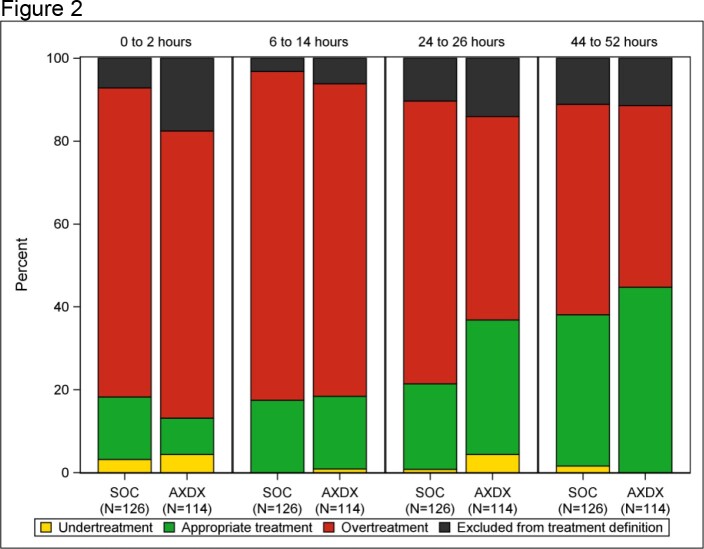

Antibiotic treatment appropriateness for antibiotic-susceptible Escherichia coli, and Klebsiella and Proteus isolates (N= 240) by time after randomization and treatment arm. Black: Excluded from treatment definition if subjects received no antibiotics, antibiotics without susceptibility results reported, or only an aminoglycoside, azithromycin, penicillin, rifaximin, or atovaquone. AXDX, rapid susceptibility testing group.

**Methods:**

Subjects enrolled in the RAPIDS GN trial who had blood cultures with monomicrobial Gram negative bacilli that were “on-panel” for the rapid test were included. Antibiotics administered from randomization (0 h) through 52 h were evaluated. Antibiotic therapy was classified based on SOC AST as: undertreatment (antibiotic to which the blood isolate was resistant), appropriate treatment (narrowest spectrum antibiotic to which the blood isolate was susceptible), and overtreatment [overly broad-spectrum antibiotic(s) based on blood isolate AST]. Antibiotic-resistant isolates were defined as 3^rd^ generation cephalosporin non-susceptible or carbapenem-resistant isolates.

**Figure:**
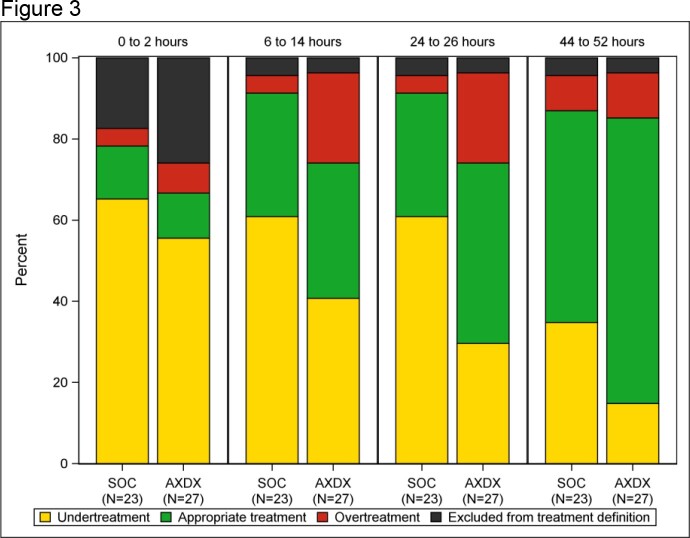

Antibiotic treatment appropriateness for antibiotic-resistant (3rd generation cephalosporin non-susceptible or carbapenem-resistant) Escherichia coli, and Klebsiella and Proteus isolates (N= 50) by time after randomization and treatment arm. Black: Excluded from treatment definition if subjects received no antibiotics, antibiotics without susceptibility results reported, or only an aminoglycoside, azithromycin, penicillin, rifaximin, or atovaquone. AXDX, rapid susceptibility testing group.

**Results:**

388/448 (87%) subjects were included (197 SOC, 191 rapid). Among susceptible isolates, between 0 through 2 h post-randomization (before AST results), overtreatment was more common for *Escherichia coli*, and *Klebsiella* and *Proteus* species than for other species (72% vs. 41%, p ≤0.001; Fig 1). Among 290 *E. coli, Klebsiella* and *Proteus* isolates, treatment appropriateness improved with time after randomization in both SOC and rapid testing groups. The proportion with appropriate treatment was higher in the rapid testing group than the SOC group at all time points after 24 h post-randomization and differed by resistance. Overtreatment was common for susceptible isolates while undertreatment was common for resistant isolates (Fig. 2 and 3). Among resistant isolates, by 52 h post-randomization undertreatment decreased to 15% in the rapid testing group but only 35% in the SOC group (Fig 3).

**Conclusion:**

In the RAPIDS GN trial, rapid AST had the greatest benefit on management of antibiotic-resistant *E. coli, Klebsiella,* and *Proteus* bacteremia. These findings can inform antibiotic stewardship and design of future blood culture diagnostic trials.

**Disclosures:**

**Ritu Banerjee, MD, Ph.D**, bioMerieux: Grant/Research Support|bioMerieux: company is providing partial support for an ongoing trial unrelated to submitted abstract **Sarah B. Doernberg, MD, MAS**, Basilea: Clinical events committee/adjudication committee participation|F2G: Grant/Research Support|Genentech: Advisor/Consultant|Gilead: Grant/Research Support|Janssen/J+J: Advisor/Consultant|Pfizer: Grant/Research Support|Regeneron: Grant/Research Support|Shinogi: Clinical events committee/adjudication committee participation **Robin Patel, MD**, Abbott Laboratories: Advisor/Consultant|Adaptive Phage Therapeutics: Grant/Research Support|Adaptive Phage Therapeutics: Mayo Clinic has a royalty-bearing know-how agreement and equity in Adaptive Phage Therapeutics.|BIOFIRE: Grant/Research Support|CARB-X: Advisor/Consultant|ContraFect: Grant/Research Support|Day Zero Diagnostics: Advisor/Consultant|HealthTrackRx: Advisor/Consultant|Mammoth Biosciences: Advisor/Consultant|Netflix: Advisor/Consultant|Oxford Nanopore Technologies: Advisor/Consultant|PhAST: Advisor/Consultant|See details: Patent on Bordetella pertussis/parapertussis PCR issued, a patent on a device/method for sonication with royalties paid by Samsung to Mayo Clinic|See details: continued, patent on an anti-biofilm substance issued|TenNor Therapeutics Limited: Grant/Research Support|Torus Biosystems: Advisor/Consultant|Trellis Bioscience, Inc.: Advisor/Consultant

